# Assessing the Quality of Life, Coping Strategies, Anxiety and Depression Levels in Patients with Long-COVID-19 Syndrome: A Six-Month Follow-Up Study

**DOI:** 10.3390/diseases12010021

**Published:** 2024-01-11

**Authors:** Adrian Vasile Bota, Felix Bratosin, Iulia Bogdan, Susa Septimiu-Radu, Adrian Cosmin Ilie, Sonia-Roxana Burtic, David Vladut Razvan, Raluca Tudor, Mirela Florica Indries, Andrei Nicolae Csep, Ariadna Petronela Fildan, Camelia Melania Budea, Iosif Marincu

**Affiliations:** 1 Methodological and Infectious Diseases Research Center, Department of Infectious Diseases, “Victor Babes” University of Medicine and Pharmacy, Eftimie Murgu Square 2, 300041 Timisoara, Romania; bota.adrian1@yahoo.com (A.V.B.); felix.bratosin@umft.ro (F.B.); iulia-georgiana.bogdan@umft.ro (I.B.); septimiu.susa@umft.ro (S.S.-R.); vladut-razvan.david@umft.ro (D.V.R.); fizedean.camelia@umft.ro (C.M.B.); imarincu@umft.ro (I.M.); 2 Doctoral School, “Victor Babes” University of Medicine and Pharmacy, Eftimie Murgu Square 2, 300041 Timisoara, Romania; dr.soniaburtic@umft.ro; 3 Department of Infectious Diseases, “Victor Babes” University of Medicine and Pharmacy, 300041 Timisoara, Romania; 4 Department III Functional Sciences, Division of Public Health and Management, “Victor Babes” University of Medicine and Pharmacy, 300041 Timisoara, Romania; ilie.adrian@umft.ro; 5 Department II, Discipline of Medical Communication, “Victor Babes” University of Medicine and Pharmacy, Eftimie Murgu Square 2, 300041 Timisoara, Romania; 6 Second Discipline of Neurology, “Victor Babes” University of Medicine and Pharmacy, Eftimie Murgu Square 2, 300041 Timisoara, Romania; 7 Department of Psycho-Neuroscience and Recovery, Faculty of Medicine and Pharmacy, University of Oradea, Str. Universitatii nr. 1, 410087 Oradea, Romania; mirela.indries@gmail.com; 8 Department of Pulmonology, Faculty of Medicine, “Ovidius” University of Constanta, 900470 Constanta, Romania; ariadnapetrofildan@yahoo.com

**Keywords:** COVID-19, post-acute COVID, long COVID

## Abstract

This longitudinal study investigates the psychosocial effects of long-COVID Syndrome, a domain still not extensively researched. It specifically evaluates the quality of life, coping mechanisms, anxiety and depression levels in COVID-19 survivors, differentiating between those with and without long-COVID Syndrome. Conducted at the Victor Babes Hospital for Infectious Diseases and Pulmonology in Timisoara, Romania, the study utilized a cohort of patients diagnosed with mild to moderate COVID-19. The following standardized tools: WHOQOL-BREF for quality of life, COPE-60 for coping strategies, and the Hospital Anxiety and Depression Scale (HADS), were employed for the assessment. The sample consisted of 86 patients displaying persistent post-acute symptoms and 432 asymptomatic patients at the 6-month post-discharge mark. Patients with frequent post-acute symptoms reported significantly higher levels of fatigue (8.2 ± 1.4), cognitive difficulties (7.5 ± 1.6), and respiratory challenges (7.8 ± 1.3), along with a markedly lower overall quality of life (7.0 ± 1.5) compared to their asymptomatic counterparts. HADS scores revealed elevated depression (6.8 ± 1.9) and anxiety (7.1 ± 2.3) in the symptomatic group. Quality of life, as evaluated through the use of WHOQOL-BREF, showed lower scores in the symptomatic cohort across physical (58.8 ± 15.8), mental (56.3 ± 16.4), and social domains (50.2 ± 17.5). COPE-60 findings indicated a higher prevalence of disengagement (56.4%) and emotion-focused coping strategies (61.8%) in the symptomatic group, in contrast to 30.1% and 37.0%, respectively, in the asymptomatic group. The study highlights that long-COVID Syndrome significantly deteriorates the quality of life and is associated with increased depression and anxiety levels. The prevalent use of disengagement and emotion-focused coping strategies among patients with persistent symptoms suggests a need for enhanced psychosocial support tailored to this subgroup.

## 1. Introduction

The coronavirus disease 2019 (COVID-19), caused by the severe acute respiratory syndrome coronavirus 2 (SARS-CoV-2), has posed unprecedented challenges to global public health and socio-economic systems [1]. Since its emergence in late 2019, millions have been infected worldwide, with significant morbidity and mortality [2]. As of 2022, COVID-19 has resulted in the death of millions globally, causing immense strain on healthcare systems and profoundly affecting the lives of those who survived [3].

While many who contract COVID-19 experience a full recovery, a significant portion have reported persistent symptoms long after their initial diagnosis [4]. Termed as “Post-COVID Syndrome” or “Long COVID”, this phenomenon encompasses a myriad of symptoms, including fatigue, joint pain, cognitive disturbances, and respiratory complications, among others [5]. Nevertheless, some symptoms are associated directly with long-COVID, while others accompany it. The exact physiological mechanism behind long-COVID syndrome remains an area of active research, but its implications for the quality of life of those affected are evident [6].

Recent studies estimate that a substantial proportion of COVID-19 survivors, ranging from 10% to 30%, may experience symptoms of long-COVID syndrome [7,8]. The persistence and unpredictability of these symptoms have led to mounting concerns about long-term health impacts, healthcare needs, and the subsequent socio-economic ramifications. This has ignited global interest in understanding the spectrum of post-acute sequelae of SARS-CoV-2 infection and identifying nterventions to mitigate these lasting effects [9,10].

The rollout of COVID-19 vaccinations has been a beacon of hope, significantly reducing infection rates, hospitalizations, and deaths in many parts of the world, with a worldwide campaign initiated at the end of 2020, during the first year of the pandemic, giving credits to the mRNA technology [11,12,13]. While vaccines are predominantly used for preventing acute COVID-19, there is growing evidence indicating that vaccination may also play a role in reducing the incidence of long-COVID symptoms in vaccinated individuals. However, the complete relationship between vaccination status and the incidence and severity of long-COVID is yet to be thoroughly understood [14,15]. Nevertheless, long-COVID is still a disputed topic, and despite the volume of research surrounding COVID-19, there remains a limited understanding of the interplay between quality of life, coping strategies, and the psychological impacts, specifically depression, in individuals diagnosed with long-COVID syndrome. Gaining insights into these areas is essential to devising holistic care strategies for affected individuals.

Therefore, the present study aims to undertake a longitudinal assessment of quality of life, coping strategies, anxiety, and depression in COVID-19 patients, drawing comparisons between those with and without long-COVID syndrome. Our hypotheses posit that individuals with long-COVID syndrome will report diminished quality of life, different coping mechanisms, and higher rates of anxiety and depression compared to their counterparts without the syndrome. Through this research, we aspire to illuminate the psychosocial dimensions of long-COVID and contribute to the collective understanding of its long-term implications.

## 2. Materials and Methods

### 2.1. Research Design and Ethical Considerations

The study employed a longitudinal research design to explore the quality of life, coping strategies, anxiety and depression among COVID-19 patients from the onset of their diagnosis and subsequently over a defined period. This approach was taken to understand the trajectory of these parameters, especially in the context of long-COVID syndrome. Patients were recruited after admission to the Victor Babes Hospital for Infectious Diseases and Pulmonology in Timisoara, Romania, affiliated with the Victor Babes University of Medicine and Pharmacy from Timisoara. Adhering to the strictest ethical standards, the research was approved by the Local Commission of Ethics for Scientific Research, which is in alignment with the EU GCP Directives 2005/28/EC, ICH guidelines, and the principles specified in the Declaration of Helsinki. Before patient inclusion, a signed informed consent was obtained from all patients willing to participate in the study after carefully explaining the study methods and objectives.

### 2.2. Inclusion Criteria and Definitions

**Figure 1:**
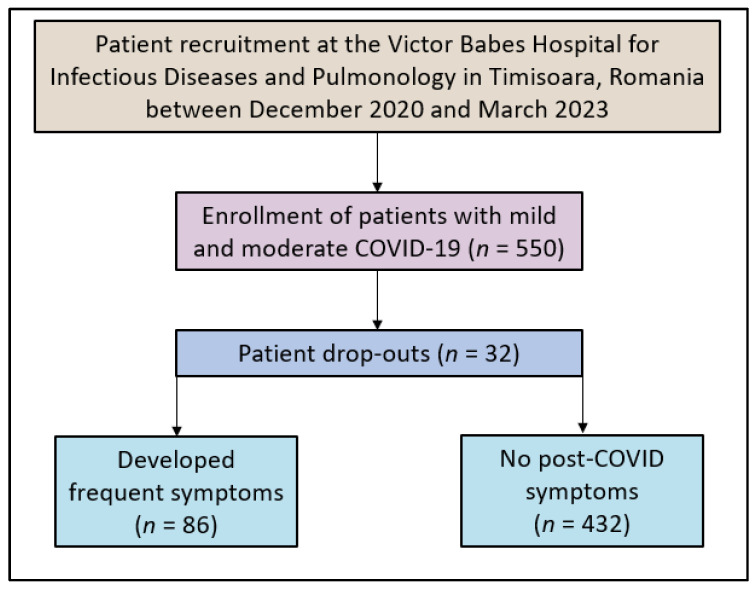
Study flowchart.

In our study, we employed a methodical and continuous selection process to recruit patients, focusing on those willing to participate, according to the study flowchart presented in Figure 1. The participant selection began by collaborating with treating physicians to identify potential candidates diagnosed with mild to moderate COVID-19. Eligible patients were adults aged 18 and above who were admitted to hospitals for their condition, ensuring the exclusion of severe cases and thereby eliminating potential confounding factors. For each month of the study, a specific number of patients were targeted to ensure a substantial sample size for the entire research duration. The exclusion criteria included patients who did not consent to participate in the study, those with a history of pre-existing chronic respiratory or psychiatric conditions, and those with incomplete questionnaire responses. Only patients after mild or moderate COVID-19 were considered for study inclusion to avoid confounding effects of severe COVID-19. The decision to assign patients to the two groups was binary: the development of frequent symptoms or disease resolution without symptoms at 6 months.

Long COVID-19 syndrome refers to a collection of symptoms that persist beyond four weeks from the onset of the initial acute symptoms of the SARS-CoV-2 virus. Despite biochemical evidence that viral replication ceases about four weeks after initial infection, some individuals continue to experience lingering symptoms. The Center for Disease Control (CDC) has formulated the term “post-COVID syndrome” to encompass long-COVID symptoms and persistent post-COVID syndrome (PPCS), multiorgan effects of COVID-19, and the impacts of COVID-19 treatment or hospitalization [16]. Common manifestations of this syndrome include fatigue, brain fog, dyspnea, autonomic dysfunction, and various symptoms related to cardiovascular, pulmonary, renal, neuropsychiatric, endocrine, and hematologic systems. The duration, severity, and specific manifestations can vary widely among patients.

COVID-19 severity was classified according to the World Health Organization (WHO) guidelines [17]. Mild COVID-19 cases are characterized by symptoms such as fever, cough, sore throat, malaise, headache, muscle pain, nausea, vomiting, diarrhea, and loss of taste and smell without evidence of viral pneumonia or hypoxia. Moderate cases involve clinical signs of pneumonia (fever, cough, dyspnea, tachypnea) but no signs of severe pneumonia, including SpO2 ≥ 90% on room air. All cases were confirmed through the use of a RT-PCR test.

### 2.3. Variables

The longitudinal assessment spanned over a 6 months period post-admission, observing the potential emergence and persistence of long-COVID syndrome. Variables assessed included the patient’s age, gender, socio-economic background, medical history, and COVID-19 severity. A paramount focus was placed on their quality of life, coping strategies, and mental health status. Through these methods, the study aimed to discern patterns and correlations that might offer insights into the evolution of long-COVID syndrome over time. All data collected were anonymized in accordance with the EU GDPR requirements.

### 2.4. Surveys Employed

In the study, a thorough approach was taken to understand the experiences of the participants, employing various validated instruments. The WHOQOL-BREF [18], consisting of 26 questions, was used to assess the overall quality of life. Additionally, to evaluate the coping strategies of patients during and post their COVID-19 illness, the COPE-60 [19] tool was introduced. The study also incorporated the Hospital Anxiety and Depression Scale (HADS) [20], which includes 14 items, to determine the levels of anxiety and depression among the participants. Furthermore, a set of specific questions was designed to gather detailed information about the patients’ experiences with COVID-19, including symptoms, hospitalization, and health status after recovery.

The COPE-60 tool is divided into various subscales, each representing different coping methods. The disengagement subscale measures avoidance coping, where individuals distance themselves from stressors or related emotions; higher scores suggest a tendency to avoid confronting stressors. The Engagement subscale evaluates an approach coping strategy, indicating how individuals actively deal with stressors; a higher score here implies a proactive approach to stress. The emotion-focused subscale focuses on managing emotional distress rather than the actual problem, with higher scores indicating a preference for strategies like seeking emotional support or expressing feelings. Lastly, the problem-focused subscale assesses direct problem-solving strategies, where higher scores mean a preference for directly addressing and resolving stressors.

### 2.5. Data Collection and Quality Control

Participants were given the aforementioned surveys upon their admission (baseline) and at a predefined interval of 6 months post their discharge. This structured approach ensured consistent tracking and assessment. Data were collated and analyzed using SPSS v.26 (SPSS Inc., Chicago, IL, USA) statistical software. Descriptive statistics were first employed to understand the demographic distribution, followed by inferential statistics to decipher potential correlations and patterns. Ensuring data integrity and consistency, double data entry methods were employed. Regular audits of the data collection process were scheduled to confirm adherence to the study protocol. Any inconsistencies or discrepancies identified during these audits were addressed promptly to maintain the quality of the study findings.

### 2.6. Statistical Analysis

The study’s data management and analysis procedures were carried out using SPSS version 26.0 (SPSS Inc., Chicago, IL, USA). The participant selection was based on a convenience sampling method, aiming for at least 180 respondents. This sample size was determined to ensure a 95% confidence level with a margin of error of 10%. In terms of data representation, continuous variables were shown as mean ± standard deviation (SD). On the other hand, categorical variables were presented through their frequencies and percentages. For the purpose of comparing two means of continuous variables, Student’s *t*-test was employed. Additionally, the Chi-square test was used for analyzing categorical variables. The study established a *p*-value of less than 0.05 as the criterion for considering results to be statistically significant (95% statistical acceptance level). All results were double-checked to ensure accuracy and reliability.

## 3. Results

In the current study, we observed a total of 86 patients presenting frequent post-acute COVID-19 symptoms and compared them to 432 individuals who reported no symptoms at 6 months after hospital discharge. Both groups were similar in age, with means of 55.2 ± 8.6 years for those with symptoms and 54.8 ± 8.9 years for the asymptomatic group (*p* = 0.786). BMI values were also comparable, averaging 24.5 ± 4.2 and 24.8 ± 4.0, respectively (*p* = 0.656). The proportion of individuals who smoked was virtually identical in both groups at around 21%. Alcohol use was reported by 51 (58.1%) of symptomatic individuals and 214 (49.5%) of those without symptoms, but this difference was not statistically significant (*p* = 0.302). Similarly, substance use and urban origins showed no significant variations between the cohorts. When considering education levels, distributions across high school, college, and university degrees were evenly matched between groups (*p* = 0.730). The percentage of individuals vaccinated against COVID-19 was slightly lower among symptomatic patients at 18 (20.9%) compared to 122 (28.2%) in the asymptomatic group, though this difference was not quite significant (*p* = 0.072). The Charlson Comorbidity Index (CCI) and initial COVID-19 severity similarly revealed no significant disparities between the two groups, as described in Table 1.

In the unstandardized survey presented in Table 2, it was observed that lingering fatigue or tiredness after COVID-19 was considerably more pronounced in the frequent symptoms group, with a mean score of 8.2, as opposed to 5.1 in the asymptomatic group (*p* < 0.001). Similarly, cognitive difficulties like brain fog or memory issues were notably higher among the symptomatic patients, scoring an average of 7.5 in sharp contrast to 5.4 in their counterparts (*p* < 0.001). Respiratory challenges after recovery also showed a significant disparity: those with frequent symptoms rated it at 6.8, while those without symptoms reported a milder impact with a score of 4.8 (*p* < 0.001). The toll on the overall quality of life post-illness was palpable among those with symptoms, scoring 7.0 compared to 3.2 in the asymptomatic group (*p* < 0.001).

Sadness, hopelessness, or depressive feelings were more predominant among the symptomatic individuals, with an average of 7.3 out of a maximum of 10, while the no-symptoms group averaged 5.9 (*p* < 0.001). Likewise, reliance on coping strategies post-recovery was higher among the symptomatic group, with a score of 6.7 against 5.0 for the other group (*p* < 0.001). Evaluations of current mental well-being in comparison to the pre-COVID period were lower among those with symptoms, scoring 4.8, as opposed to 6.5 in the no-symptoms cohort (*p* < 0.001). Patients with frequent symptoms also reported persistent symptoms related to organs post-recovery (*p* < 0.001). Interestingly, when questioned about feeling supported in managing lingering symptoms or challenges post-COVID-19, those with symptoms felt less supported, scoring 5.6, whereas the no-symptoms group felt more supported with a score of 7.5 (*p* < 0.001). Lastly, the experience of COVID-19 seemed to have a more profound influence on the health and wellness perspective of those with frequent symptoms.

Another key focus was to evaluate anxiety and depression levels among individuals who reported frequent post-acute COVID-19 symptoms and compare them with those who did not manifest any symptoms post-infection. The assessment was facilitated using the HADS survey, as presented in Table 3. Upon examination of the data, patients with frequent symptoms demonstrated notably higher levels of anxiety with an average score of 7.1 ± 2.3, in contrast to those without symptoms, who reported a mean score of 5.5 ± 3.6 (*p* < 0.001). The trend was similar in terms of depression levels, where those experiencing frequent symptoms had a mean score of 6.8 ± 1.9, significantly higher than the 5.1 ± 2.4 average of the asymptomatic group (*p* < 0.001). When the total scores, indicative of overall mental distress, were considered, there was a clear disparity between the two groups. Those with frequent post-acute symptoms had a mean total score of 13.4 ± 4.7, while the group without symptoms averaged 10.6 ± 4.8 (*p* < 0.001).

The WHOQOL-BREF survey was employed to evaluate the quality of life in patients presenting with frequent post-acute COVID-19 symptoms and compare it to those without subsequent symptoms after SARS-CoV-2 infection. The results revealed a significant divergence in the quality of life between the two groups across multiple domains. In the physical domain, patients with frequent post-acute symptoms scored notably lower, with an average of 58.8 ± 15.8, as opposed to those without symptoms, who achieved an average score of 67.5 ± 16.7 (*p* = 0.002). The difference was also evident in the mental domain, where individuals experiencing frequent symptoms averaged 56.3 ± 16.4, contrasting the 64.0 ± 15.9 average of the group devoid of post-COVID symptoms (*p* = 0.004).

Further differences between the groups emerged in the social domain, with the frequent symptoms cohort scoring 50.2 ± 17.5, significantly lower than the 59.5 ± 18.0 average of their counterparts (*p* = 0.002). However, when assessing the environmental domain, while those with symptoms had a lower score (57.0 ± 14.8) compared to those without symptoms (62.4 ± 17.2), the difference was not statistically significant at the conventional threshold, with a *p*-value of 0.056, as seen in Table 4.

The most pronounced divergence was seen in the disengagement coping strategy. A striking 62 (72.1%) of those with ongoing symptoms scored above the median, a substantial increase compared to the 130 (30.1%) observed in the group without symptoms (*p* < 0.001). These data strongly suggest that patients grappling with continued health issues tend to retreat from stressors more than those who have fully recovered. Regarding emotion-focused coping, 68 (79.1%) of those with symptoms were above the median, indicating a predominant use of emotional management to cope with stress. This is a significant contrast to the 162 (37.5%) in the asymptomatic group (*p* < 0.001), underscoring a reliance on emotional coping mechanisms among patients with persistent symptoms.

Conversely, problem-focused coping was less prevalent among those with enduring symptoms, with only 24 (27.9%) scoring above the median, as opposed to 186 (43.1%) of those without symptoms (*p* = 0.009). This suggests that patients free from post-acute symptoms are more inclined to confront stressors head-on, seeking to address or mitigate them actively. Engagement coping strategies exhibited no significant difference statistically, with 36 (41.8%) of symptomatic individuals and 198 (45.8%) of those without symptoms scoring above the median (*p* = 0.498), as described in Table 5.

The quality of life domains, particularly the mental and physical domains of the WHOQOL-BREF, exhibit a strong negative association with the HADS total score, with coefficients of −0.296 and −0.247, respectively, both reaching statistical significance (*p* < 0.001 and *p* = 0.004). This suggests that better-perceived quality of life in these domains is associated with lower levels of anxiety and depression among patients. Additionally, coping strategies present contrasting effects, while disengagement shows a positive association with higher HADS scores (coefficient: 0.298, *p* < 0.001), indicating that reliance on disengagement strategies might exacerbate mental health challenges. Engagement strategies show a negative association (coefficient: −0.103, *p* = 0.046), implying their potential benefit in mitigating anxiety and depression. Furthermore, the presence of lingering fatigue and cognitive difficulties post-COVID are significantly associated with higher HADS scores (coefficients: 0.405 and 0.348, both *p* < 0.001), highlighting the substantial mental health impact of these persistent symptoms, as presented in Table 6.

## 4. Discussion

### 4.1. Important Findings and Literature Review

The COVID-19 pandemic has, undoubtedly, posed an array of medical and psychological challenges to individuals globally. The findings from our study underscore the profound disparities in the lived experiences and outcomes between individuals suffering from frequent post-acute COVID-19 symptoms and those who were cured of COVID-19 without significant long-term complications, both mentally and physically. Similarly, other large studies suggest that people with mental conditions are at significant risk for deterioration [21].

One of the most compelling revelations of the current study pertains to the significant variance in post-illness quality of life between the two cohorts. Despite both groups being statistically similar in terms of demographics, comorbidities, initial disease severity, and other factors, the symptomatic group reported markedly more pronounced fatigue, cognitive difficulties, respiratory issues, and an overall deteriorated quality of life. These findings resonate with the broader understanding that post-COVID symptoms can have a debilitating effect on individuals, extending well beyond the acute phase of the disease and increasing the frequency of headaches, fatigue, dyspnea, and other symptoms that can influence the quality of life, as previously reported [22].

Depressive sentiments and feelings of sadness were substantially more prominent among those experiencing frequent symptoms, further accentuating the psychological toll of long-COVID syndrome, similar to what was recently described, where approximately 35% of all patients with long-COVID syndrome experienced depressive symptoms [23]. In our study, the elevated reliance on coping strategies in this group suggests a greater need to manage persistent distress and challenges, further compounded by their reported sentiment of feeling less supported in handling their ongoing symptoms.

The more nuanced exploration into the mental health of these patients revealed discernible disparities in anxiety and depression levels. The HADS survey outcomes underscored the heightened levels of both anxiety and depression among patients with frequent post-acute symptoms. While the causality cannot be decisively concluded from our study, it is plausible that the continuous struggle with lingering symptoms may amplify feelings of distress, anxiety, and desolation.

Quality of life, as assessed by using the WHOQOL-BREF survey, further elucidated the gap between the two groups. The symptomatic group consistently scored lower across multiple domains, emphasizing the pervasive impact of long-COVID syndrome on the physical, mental, and social well-being of affected individuals. Notably, the environmental domain, which reflects an individual’s satisfaction with their living conditions, physical safety, and accessibility to resources, showed only a marginal difference between the groups, suggesting that the primary disparities are rooted in personal health experiences rather than external environmental factors. Other studies found that both younger and older adults experienced significantly reduced quality of life satisfaction, most significantly on the physical level [24,25].

Intriguingly, the COPE-60 survey offered insights into the varied coping mechanisms employed by the two groups. The symptomatic cohort’s proclivity towards disengagement and emotion-focused coping hints at a potential strategy of distancing themselves from the distress or managing it through emotional outlets. In contrast, the asymptomatic group seemed more inclined towards problem-focused coping, suggesting a more proactive approach to challenges. This divergence in coping strategies reiterates the diverse psychological responses elicited by the aftermath of COVID-19 and highlights the need for individualized therapeutic interventions tailored to the unique needs and experiences of patients.

Even though the current study used three different instruments to evaluate quality of life and psychological impact of long-COVID, other studies used the EuroQol or the SF-36, and found that the EuroQol dimension and the SF-36 questionnaire both indicated significant impairments in long-COVID patients in the dimensions of “usual activities”, “pain/discomfort”, and “anxiety/depression” [26,27]. Another study found the “self-care” dimension remained largely unaffected, while there was also a varied EQ VAS value distribution, emphasizing differences in symptom burden and quality of life impacts [28]. The EQ-5D-5L index score closely matched the results from patients six months post COVID-19 related acute respiratory distress syndrome [29].

Another QOL instrument used in different studies assessing the quality of life among patients with long-COVID syndrome is the EQ-VAS questionnaire, where 59% of individuals recovering from COVID-19 experienced a diminished quality of life as measured via the EQ-VAS survey. Additionally, according to the EQ-5Q-5L survey, 42% reported pain/discomfort, 38% faced anxiety/depression, 36% encountered mobility issues, 28% had difficulties with routine tasks, and 8% struggled with self-care post-recovery [30].

To assess the effects of prevalent respiratory symptoms like dyspnea, with a 60% prevalence in one recent study that focused on HRQoL, the SGRQ was used [28]. Respiratory symptoms heavily impacted patients’ PA, yielding scores resembling those of COPD patients with moderate disease severity. The symptom scores for long-COVID were, however, lower than any GOLD stage group. In terms of gender analysis, female patients reported lower SGRQ activity scores, EQ-5D-5L index values, and various impairments on the SF-36. Two-thirds of female participants experienced six or more symptoms compared to one-third of male participants, suggesting a potentially greater symptom intensity or range for women. Even though the current study did not find significant differences, possible explanations might include gender disparities in symptom experiences or coping mechanisms during stress [31].

In our study, the selection of participants with persistent post-COVID symptoms naturally predicted higher physical symptom scores. However, the significant differences observed in psychological variables, which were not a basis for selection, underscore the multifaceted impact of long-COVID. This finding highlights the need for a holistic understanding and approach to long-COVID that encompasses both physical and mental health dimensions.

### 4.2. Study Limitations

While the present study provides valuable insights into the quality of life, coping strategies, and depression among COVID-19 patients, particularly highlighting differences between those with and without long-COVID syndrome, there are some notable limitations to consider. First, the sample population was exclusively sourced from one clinic in Romania, potentially limiting the generalizability of the findings to broader populations and settings. Second, by exclusively including individuals with mild to moderate COVID-19, the study inherently excludes the perspectives of those with severe cases, making it challenging to understand the full spectrum of post-COVID implications. Additionally, the use of self-reported surveys, although based on established tools, might be subject to participant recall bias, especially considering the emotional and physical strain of the disease. The convenience sampling method, while practical, may not yield a sample representative of the broader COVID-19 survivor population. Lastly, patient assessment after 6 months post-discharge may not be sufficient to capture longer-term implications of long-COVID syndrome.

## 5. Conclusions

The impact of the COVID-19 pandemic extends beyond its immediate clinical implications, as evidenced by the stark contrasts in post-illness experiences between those with and without lingering symptoms. This study highlights that individuals suffering from frequent post-acute COVID-19 symptoms face pronounced challenges in terms of cognitive and physical difficulties, notably fatigue and respiratory issues, which significantly deteriorate their overall quality of life. Additionally, there is a salient psychological burden, manifested in heightened depressive sentiments and elevated levels of anxiety and depression. This mental toll is further accentuated by the symptomatic group’s reliance on coping mechanisms like disengagement and emotion-focused strategies, suggesting a potential inclination towards avoidance or emotional regulation in the face of persistent distress. In contrast, those without frequent symptoms exhibit proactive problem-focused coping. These findings underscore the pressing need for comprehensive post-COVID care that not only addresses physical sequelae but also offers tailored psychological support, recognizing the varied challenges and coping mechanisms employed by affected individuals.

## Figures and Tables

**Table 1:** Background characteristics of patients assessed with frequent post-acute COVID-19 symptoms and those with no symptoms after SARS-CoV-2 infection.

	Frequent Symptoms (*n* = 86)	No Symptoms (*n* = 432)	*p*-Value *	Effect Size
Age, years	55.2 ± 8.6	54.8 ± 8.9	0.786 **	0.002
BMI	24.5 ± 4.2	24.8 ± 4.0	0.656 **	0.090
Currently smoking	18 (20.9%)	91 (20.8%)	0.988	0.006
Alcohol use (occasionally)	51 (58.1%)	214 (49.5%)	0.302	0.001
Substance use	8 (9.3%)	38 (8.8%)	0.908	0.835
Place of origin (urban)	36 (65.1%)	206 (70.8%)	0.455	0.038
Education			0.730	
High school	24 (27.9%)	98 (22.7%)		0.039
College	40 (46.5%)	206 (47.7%)		0.003
University	22 (25.6%)	128 (29.6%)		0.027
COVID-19 vaccinated	18 (20.9%)	122 (28.2%)	0.072	0.055
CCI > 2	16 (18.6%)	80 (18.5%)	0.490	0.001
COVID-19 severity			0.526	0.074
Mild	60 (69.8%)	256 (59.3%)		
Moderate	26 (30.2%)	176 (40.7%)		

* Chi-square or Fisher’s exact test; ** Student’s *t*-test; SD—Standard Deviation; BMI—Body Mass Index; CCI—Charlson Comorbidity Index. The effect size is described as “r” for the continuous variables and Cramer’s V for the Chi-square tests.

**Table 2:** Unstandardized survey results to assess long-COVID symptoms and complications.

Questions (Answers Given on a Scale from 1 to 10)	Frequent Symptoms (*n* = 86)	No Symptoms (*n* = 432)	*p*-Value *	Effect Size
How frequently have you experienced lingering fatigue or tiredness after your COVID-19 diagnosis?	8.2 ± 3.4	5.1 ± 1.5	<0.001	0.534
To what extent do you experience cognitive difficulties (e.g., brain fog or memory issues) since your illness?	7.5 ± 2.6	5.4 ± 1.3	<0.001	0.471
How often have you felt short of breath or faced respiratory challenges after recovering from the acute phase of the disease?	6.8 ± 1.3	4.8 ± 2.2	<0.001	0.326
In the aftermath of your illness, how would you rate the impact of COVID-19 on your overall quality of life?	7.0 ± 1.5	3.2 ± 1.4	<0.001	0.677
How frequently have feelings of sadness, hopelessness, or depression affected your daily life post-COVID-19?	7.3 ± 2.2	5.9 ± 1.6	<0.001	0.305
Since SARS-CoV-2 infection, how often have you been using negative coping strategies to manage post-COVID symptoms or mental distress?	6.7 ± 3.1	5.0 ± 1.5	<0.001	0.330
How would you evaluate your current mental well-being compared to the period before your COVID-19 diagnosis?	4.8 ± 1.6	6.5 ± 2.7	<0.001	0.233
Have you noticed any persistent symptoms related to your heart, kidneys, or other organs after your initial recovery?	6.9 ± 2.4	4.5 ± 1.4	<0.001	0.486
How supported do you feel in managing any lingering symptoms or mental challenges post-COVID-19?	5.6 ± 1.6	7.5 ± 3.3	<0.001	0.243
To what degree do you believe that your experiences post-COVID-19 have influenced your overall perspective on health and wellness?	7.3 ± 1.2	5.4 ± 1.7	<0.001	0.428

* Data analyzed using Student’s *t*-test; Data presented as mean ± standard deviation of the 10-point scale questionnaire.

**Table 3:** HADS survey results stratified by patients with frequent post-acute COVID-19 symptoms and those with no symptoms after SARS-CoV-2 infection.

HADS (Mean ± SD)	Frequent Symptoms (*n* = 86)	No Symptoms (*n* = 432)	*p*-Value	Effect Size
Anxiety	7.1 ± 2.3	5.5 ± 3.6	<0.001	0.178
Depression	6.8 ± 1.9	5.1 ± 2.4	<0.001	0.259
Total score	13.4 ± 4.7	10.6 ± 4.8	<0.001	0.228

SD—Standard Deviation; SF-36—Short Form Survey (higher scores indicate higher levels of anxiety or depression).

**Table 4:** WHOQOL-BREF survey results stratified by patients with frequent post-acute COVID-19 symptoms and those with no symptoms after SARS-CoV-2 infection.

WHOQOL-BREF (Mean ± SD)	Frequent Symptoms (*n* = 86)	No Symptoms (*n* = 432)	*p*-Value	Effect Size
Physical domain	58.8 ± 15.8	67.5 ± 16.7	0.002	0.078
Mental domain	56.3 ± 16.4	64.0 ± 15.9	0.004	0.195
Social domain	50.2 ± 17.5	59.5 ± 18.0	0.002	0.172
Environmental domain	57.0 ± 14.8	62.4 ± 17.2	0.056	0.061

SD—Standard Deviation; WHOQOL-BREF—Brief Version of the World Health Organization Quality of Life survey (higher scores indicate better quality of life).

**Table 5:** COPE-60 survey results stratified by patients with frequent post-acute COVID-19 symptoms and those with no symptoms after SARS-CoV-2 infection.

Variables (% of Scores above Median)	Frequent Symptoms (*n* = 86)	No Symptoms (*n* = 432)	*p*-Value	Effect Size
Disengagement	62 (72.1%)	130 (30.1%)	<0.001	0.318
Engagement	36 (41.8%)	198 (45.8%)	0.498	0.024
Emotion Focused	68 (79.1%)	162 (37.5%)	<0.001	0.306
Problem Focused	24 (27.9%)	186 (43.1%)	0.009	0.109

SD—Standard Deviation; GAD—General Anxiety Disorder (higher scores indicate higher anxiety symptoms); COPE—Coping Orientation to Problems Experienced Inventory (higher scores indicate that patients are more likely to use a certain domain of coping strategies).

**Table 6:** Predictors of anxiety and depression: analyzing the impact of quality of life, coping strategies, long-COVID symptoms, and background characteristics based on HADS total score.

Variable	Coefficient	Std. Error	*p*-Value
Quality of Life (WHOQOL-BREF)			
-Physical Domain	−0.247	0.082	0.004
-Mental Domain	−0.296	0.073	<0.001
-Social Domain	−0.195	0.088	0.029
-Environmental Domain	−0.147	0.096	0.107
Coping Strategies (COPE-60)			
-Engagement	−0.103	0.051	0.046
-Disengagement	0.298	0.063	<0.001
-Emotion Focused	0.205	0.054	0.001
-Problem Focused	−0.053	0.038	0.168
Long-COVID Symptoms			
-Lingering Fatigue	0.405	0.098	<0.001
-Cognitive Difficulties	0.348	0.087	<0.001
-Respiratory Challenges	0.251	0.105	0.019
Background Characteristics			
-Age	−0.012	0.019	0.543
-BMI	0.024	0.027	0.376
-Smoking Status	0.149	0.203	0.435
-COVID-19 Severity	0.247	0.116	0.032
Constant	5.503	0.495	<0.001

WHOQOL-BREF—Brief Version of the World Health Organization Quality of Life survey (higher scores indicate better quality of life; BMI—Body Mass Index; COPE—Coping Orientation to Problems Experienced Inventory (higher scores indicate that patients are more likely to use a certain domain of coping strategies); Adjusted R-squared = 0.453.

## Data Availability

The data presented in this study are available upon request from the corresponding author.
